# Happy hamsters? Enrichment induces positive judgement bias for mildly (but not truly) ambiguous cues to reward and punishment in *Mesocricetus auratus*

**DOI:** 10.1098/rsos.140399

**Published:** 2015-07-29

**Authors:** Emily J. Bethell, Nicola F. Koyama

**Affiliations:** School of Natural Sciences and Psychology, Liverpool John Moores University, Byrom Street, Liverpool L3 3AF, UK

**Keywords:** animal welfare, judgement bias, emotion, environmental enrichment, psychological wellbeing, Syrian hamster

## Abstract

Recent developments in the study of animal cognition and emotion have resulted in the ‘judgement bias’ model of animal welfare. Judgement biases describe the way in which changes in affective state are characterized by changes in information processing. In humans, anxiety and depression are characterized by increased expectation of negative events and negative interpretation of ambiguous information. Positive wellbeing is associated with enhanced expectation of positive outcomes and more positive interpretation of ambiguous information. Mood-congruent judgement biases for ambiguous information have been demonstrated in a range of animal species, with large variation in the way tests are administered and in the robustness of analyses. We highlight and address some issues using a laboratory species not previously tested: the Syrian hamster (*Mesocricetus auratus*). Hamsters were tested using a spatial judgement go/no-go task in enriched and unenriched housing. We included a number of controls and additional behavioural tests and applied a robust analytical approach using linear mixed effects models. Hamsters approached the ambiguous cues significantly more often when enriched than unenriched. There was no effect of enrichment on responses to the middle cue. We discuss these findings in light of mechanisms underlying processing cues to reward, punishment and true ambiguity, and the implications for the welfare of laboratory hamsters.

## 1. Introduction

Accurate assessment of animal emotions is an important goal of welfare science [1,2] and central to the refinement of the use of animals in scientific research [3,4]. In the last decade, methods used to assess the link between affective state and cognitive processes in humans [5,6] have been successfully adapted and applied to animals. The judgement bias model of animal welfare [7–10] argues that emotion states may be measured in animals, as they are in humans, as a function of altered cognitive judgements about the likely rewarding or punishing nature of ambiguous stimuli. The value of judgement bias measures over traditional physiological and behavioural approaches is their potential to distinguish emotional valence (positive versus negative emotions). Given the large number of laboratory rodents used each year, it is important to develop proxy measures of affective state to aid in welfare assessment. Thus far, judgement bias studies on laboratory rodents have focused on rats (e.g. [7]), with two studies on mice [11,12] and none on hamsters.

Judgement bias in animals has been most often measured using a ‘go/no-go’ task in which animals are trained to discriminate between a ‘positive’ and a ‘negative’ cue and then tested on their responses to ambiguous cues [7]. Ambiguous cues possess characteristics intermediate to both the rewarded and non-rewarded stimuli, and it is responses (‘go’ or ‘no-go’) to these that quantify judgement bias. Recent studies with animals including rats [7,13–16], mice [11,12], cats [17], dogs [18–24], dairy calves [25,26], sheep [27–38], chickens [39–42], honeybees [43], rhesus macaques [44], marmosets [45], starlings [46], pigs [47–49], horses [50] and goats [51] demonstrate that emotion-mediated judgement biases may be detected using species-specific variants of the go/no-go task. So far, studies have revealed that manipulations presumed to create a negative state (such as disrupted housing conditions [7] or dehorning in calves [26]) lead to reduced responses (more ‘no-go’s) to the ambiguous probes. This negative shift in judgement bias is presumed to arise from a negative shift in underlying emotion state. Positive manipulations (e.g. addition of environmental enrichment [46]) generally lead to increased responses (more ‘go’s) at one or more of the ambiguous probes. This is presumed to arise from positive shifts in judgement bias and underlying emotion state.

A criticism of the go/no-go approach (discussed more fully in [9]) is that results may not reflect judgements about ambiguity, but instead reveal changes in motivation (due to satiation or learning), arousal or activity (due to treatment effects or task fatigue). A solution to this is to include control trials and a variable reinforcement ratio (VRR) to maintain motivation and reduce learning effects. Triangulating judgement bias with traditional measures of affect (e.g. open field (OF) test and light–dark emergence (LDE) tests) and recording behaviour during testing would allow treatment effects and task fatigue to be considered.

The active choice task is an alternative to the go/no-go task. The response (‘go’ or ‘go’) is the same to both learned stimuli, providing a built-in control for arousal effects. The active choice test has been validated with rats [52–64], starlings [65,66], pigs [67], chickens [68], grizzly bear [69] and capuchin monkey [70]. Results from these studies are varied, and several report arousal effects (e.g. [67,68]). Care must be taken to ensure that the perceived affective difference between the two reinforcers is sufficiently great to detect any shift in judgement bias [9]. For example, in the study of Parker *et al.* [64], the ‘positive’ cue signalled two pellets and the ‘negative’ cue signalled one pellet. In this case, repeated training might more rapidly lead to satiation, a decrease in reward value or reduced ability to distinguish between high and low reward. A variant of the active choice task is to use negative reinforcement, e.g. electric shock [52,55–57,59–63], but ethical issues and confounding effects of fear and stress make this method unsuitable for welfare assessment. Results from studies using negative reinforcement, which tends to be pharmacological manipulations conducted in rats, support the judgement bias model of emotion–cognition interaction in non-human animals.

Both approaches are susceptible to a number of limitations. These include: (i) cueing effects (e.g. odour cues from food), (ii) the contribution of other aspects of cognition such as risk-taking behaviour or attention for threat cues, (iii) learning the reward value of the ambiguous probes, (iv) lack of a consistent and robust statistical approach, and (v) a lack of consistency in *a priori* predictions about the direction of change in bias and associated emotion state (for example, increased responding to probes in the ‘stress’ condition is interpreted as indicating relief following the termination of the stressor in Doyle *et al.* [27] and Sanger *et al.* [31]). These limitations are rarely addressed in study design, but failure to do so may lead to misinterpretation of results.

Of the two task designs, the spatial go/no-go task has been the most widely validated and applied across species. Within Mendl *et al.*'s [10] integrative framework, emotional valence is directly associated with approach towards reward (associated with excitement and happiness) and avoidance of (or inhibition of response to) threat or punishment (associated with fear and anxiety). These underlying associations may facilitate training on the ‘go/no-go’ task (compared to learning to discriminate high versus low reward in the active choice task), making go/no-go an ideal method to develop a judgement bias task in a new species. Both tasks have been developed using auditory cues (e.g. [48,67]) and visual cues (e.g. [44,70]). In addition, the go/no-go task has been validated with spatial cues (e.g. [7,39,50]), tactile cues (e.g. [53,58]) and odour cues (e.g. [43]). Among rodents, spatial cues [7,13–16] and auditory tones [52,55–57,60–63] have been used successfully with rats, while spatial cues [39,41] and odour cues [12] have been used successfully with mice. Spatial cues are therefore suitable for first development of a task with a new species of rodent.

For the purposes of welfare assessment, the addition of environmental enrichment has been the most common positive manipulation in judgement bias studies. The majority of studies where enrichment was added reported a positive shift in judgement bias. Studies have differed in the type of enrichment added (e.g. increased space, perches, shelters, foraging substrate [44,46]), the duration of treatment (e.g. 2 h in grizzly bears [68], one week in rats [16,53], 10 days in rhesus macaques [44]), whether enrichment is removed (starlings [46]) and whether a switch in housing occurs, for example from enriched to basic and back to enriched (starlings [66] and pigs [48]). There are some exceptions: no judgement bias shift was detected in laying hens [40]; however, this study used a between-subjects design and the individual differences in fearfulness, body condition and neophobia appeared to have a greater impact on performance than the enrichment treatment. Of the four studies that employed a switch in housing conditions [46,48,65,66], three detected a treatment-induced shift in judgement bias [46,48,65]. In starlings, a pessimistic bias was noted following a reduction in enrichment but no change was noted following addition of enrichment [46]; however, a later study using similar enrichment items and increased space found an optimistic bias in enriched cages [65]. Both these studies used a cross-over design with half the subjects experiencing enriched then unenriched housing and the other half unenriched then enriched housing. Douglas *et al.* [48] also used a cross-over design in their study of pigs, with half experiencing the switch enriched-to basic-to enriched and the other half the opposite housing manipulation. Pigs currently in enriched housing interpreted the ambiguous cues more optimistically. Moreover, there was an interaction between current and past environment: pigs that were first in enriched housing reacted more negatively when subsequently housed in a barren environment. In the fourth study involving a switch in housing conditions [66], individual differences in stereotypies were found to predict pessimism. These studies highlight the importance of within-subjects study design, appropriate enrichment to create a shift of sufficient magnitude that is not masked by individual differences, and using a cross-over design to control for order effects.

Here, we aim to validate a judgement bias test for use in hamsters (*Mesocricetus auratus*) and to tackle the above criticisms within spatial judgement bias paradigm [9,13].

We measured judgement bias using a balanced, cross-over design [48] using changes in enrichment to validate the task. Hamsters were initially housed in standard laboratory cages with basic enrichment and then received highly enriched housing (E) first or experienced removal of some of the basic enrichment (R). Enrichment devices were selected according to published data from hamster preference tests and natural behaviour [71–73]. A week later hamsters moved to the opposite housing conditions allowing comparison of the two groups (ER or RE). In response to our critique above we proceeded as follows. (i) We excluded olfactory cueing effects by introducing a VRR during training. (ii) We statistically controlled for confounding effects of arousal, by including day and block of testing in our model (see below), and controlling for other aspects of cognition by triangulating data with traditional tests of affect in rodents such as OF and LDE [74], neophobia [75] tests and recording in-trial behaviours traditionally used to measure emotion state or arousal in laboratory rodents (e.g. [7,16,73–77]). (iii) To reduce the likelihood of hamsters learning that probes were never rewarded we used a VRR during training, and included unreinforced control trials at the positive and negative stimulus locations during testing. (iv) We applied a robust information-theory approach (multi-model inference) using linear mixed effects models [78–81] to assess the relative contribution of different variables to responses on the task. Generalized linear mixed models incorporate random effects, test fixed effects using generalized least squares results in more powerful tests, handle non-normal data by using link functions and exponential family distributions (e.g. normal, Poisson or binomial), and are able to handle missing data and unequal spacing over time [78,79]. Linear mixed effects models are beginning to appear in judgement bias studies (e.g. [22,51,82]) and provide a robust and flexible approach to analysis that will aid interpretation of output in light of potential confounding factors. (v) We predicted that the addition of enrichment would be associated with a positive affective state and corresponding optimistic judgement bias, while the removal of enrichment would be associated with a more negative affective state and judgement bias.

## 2. Material and methods

### 2.1 Study animals and housing

Thirty captive-born male Syrian hamsters (*Mesocricecitus auratus*) took part in the study. Hamsters were weaned at 21 days and retained in eight same-sex sibling groups throughout the study. Hamsters were 8 weeks old at the start of the study and 13 weeks old by the end of the study. The study was conducted with one cohort of hamsters in 2011 (*n*=17) and a second cohort in 2013 (*n*=13). In both years, sibling groups were housed in the same style of caging (56×38×22 cm) in the same air-conditioned room kept at a constant 20–24°C on a 12 L : 12 D cycle, with humidity maintained between 45 and 70%. Hamsters had *ad libitum* access to water and dry rodent pellets (Eurodent Diet 22% 5LF5, PMI Nutrition International Plc) and were handled twice per week from weaning for routine cage cleaning and health inspections by care staff. All animals that completed the study were assessed by care staff as healthy throughout.

### 2.2 Apparatus

Training and testing sessions were conducted in a high-sided blue opaque plastic arena (100×80×85 cm) in the housing room during the first hours of the active (dark) phase using low level light. The arena had five holes drilled along one wall, with attachments on the outside so that on each trial a drinker could be attached at any location with only the spout protruding into the arena. Three drinkers were used during the experiment. Two drinkers were used for training and maintenance during testing: one drinker contained 0.3 M sugar water and one drinker contained 0.01 M quinine hydrochloride (QHCl) solution. A third drinker was used for experimental trials (‘control’ and ‘probe’ trials) and was kept empty throughout.

### 2.3 Habituation

**Figure 1.**
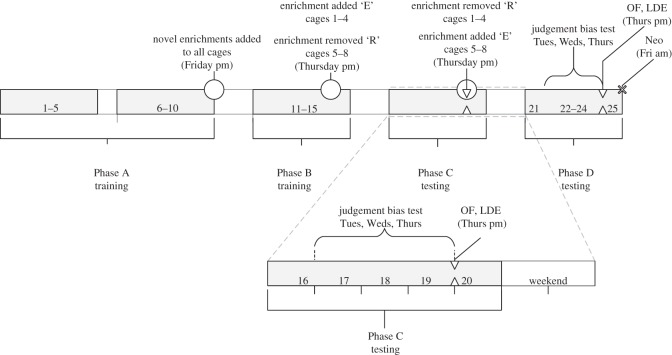
Timeline for the study, conducted over five weeks. Day numbers are shown: discrimination training (days 1–15); judgement bias testing (days 17–19 and 22–24; maintenance trials) days 16, 20 and 21). ○, Enrichment manipulation; additional behavioural tests—△, OF and LDE tests (days 19 and 24) and ×, neophobia test (Neo) (day 25).

The timeline for the study is shown in figure 1. Initially, all animals were familiarized with the arena. One familiarization session was conducted on each of four days (Tuesday–Friday) for each sibling group. The sibling group was placed in the arena with scattered food and a bottle containing sugar water attached at the rewarded location (left or right) for that group. Animals were monitored for 5 min to ensure all animals had drunk from the drinker. At the end of each daily session, the number of faecal pellets left in the arena was counted.

### 2.4 Training

#### 2.4.1 Phase A (weeks 1 and 2: days 1–10)

**Figure 2.**
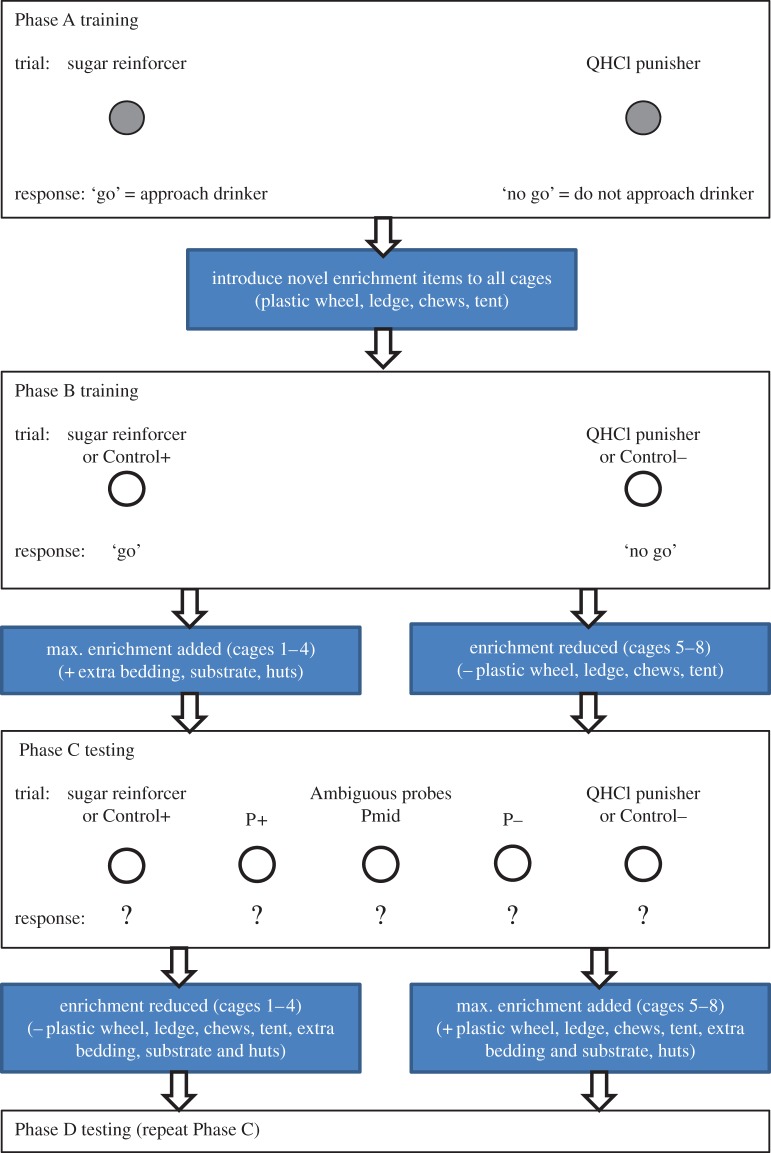
The judgement bias task. During training (Phase A) hamsters learned to approach the sugar drinker (‘go’) and not to approach (‘no go’) the QHCl drinker. At the end of Phase A, all animals received a basic provision of novel enrichment items. Training on the go/no-go task was maintained in Phase B with the inclusion of the empty drinker on some trials to create a 50% VRR (empty drinker trials: ‘Control +’ and ‘Control −). Towards the end of Phase B half of the sibling groups received the maximum complement of enrichment, while the novel enrichment items were removed from the other half of the sibling groups. Animals were tested on the judgement bias task on the following week (Phase C) with the presentation of the empty drinker at all five locations, including the three ambiguous intermediate probe locations. Enrichments were then switched and animals tested again in Phase D.

We employed a spatial judgement task used previously ([13], figure 2). In brief, hamsters were trained to approach a drinker at one location (left or right) to obtain a reward (‘go’ towards sugar water), and to refrain from approaching a drinker at the other location (right or left) to avoid an aversive liquid (‘no go’ towards QHCl). A trial began when the hamster was placed at a start point at the centre back of the arena. The trial ended when the hamster approached the drinker, or after a predetermined number of seconds, whichever occurred sooner. On the first day of Phase A, each hamster took part in 8×120 s trials. As hamsters learned the task and became faster to approach, sessions were sequentially adjusted to 8×60 s trials and then 10×30 s trials. This adjustment was made according to individual response latencies so that trial duration was reduced quickly for fast-responding hamsters, and more slowly for hamsters that needed the extra time to approach. If the hamster approached to within 3 mm of the drinker in the predetermined trial duration this was scored as a ‘go’. If the hamster did not approach the drinker in the predetermined trial duration this was scored as a ‘no-go’. An equal number of sugar and QHCl trials were run within each daily training session for each hamster. The first and last trials were always sugar trials so that each session began and ended with a positive association with the arena (same throughout all subsequent phases). A hamster was removed once he had finished drinking. Criterion for learning the discrimination task was average latency to approach the sugar drinker being shorter than average latency to approach the QHCl drinker on each of three consecutive days.

#### 2.4.2 Phase B (week 3; training days 11–15)

Training continued as for Phase A with the introduction of control trials to create a 60% variable reinforcement ratio. A control trial was a trial on which the empty drinker was presented at either of the two trained (sugar/QHCl) locations. These trials were included to ensure animals were responding to drinker location and not to odour cues. A daily training session in Phase B comprised 16×30 s trials divided into two blocks (table 1). Block 1 contained nine trials: five reinforcement trials (2 sugar : 2 QHCl, plus one) pseudorandomized with four control trials (2C+ : 2C−). Block 2 comprised a total of seven trials: three reinforcement trials (1 sugar : 1 QHCl, plus one) pseudorandomized with four control trials (2C+ : 2C−). Care was taken to remove the hamster as soon as he reached the empty drinker on control trials to reduce the possibility of animals becoming frustrated if left to attempt to drink from an empty drinker.

**Table 1.** The blocked design for training in Phase B and testing in Phases C and D. Each daily session began (block 1: trials 1 and 2) with a rewarding sugar trial followed by a QHCl trial to remind hamsters of the task contingencies. Each daily session ended (block 2: trial 16) with a sugar trial to end on a positive association and encourage maintained performance over subsequent days. Order of all other trials was pseudorandomized within each of block. S+=Sugar; Q−=QHCl; C+=Control at sugar location; C−=Control at QHCl location; P+=ambiguous probe closest to the sugar location; Pmid=middle probe; P−=ambiguous probe closest to the QHCl location. Trial numbers are shown in parentheses.

	block			
Phase	1		2	
B (trial n)	S+, Q− (1, 2)	2C+, 2C−, S+, Q− (3–9)	2C+, 2C−, S+, Q− (10–15)	S+ (16)
C/D (trial n)	S+, Q− (1, 2)	C+, C−, P+, Pmid, P− S+, Q− (3–9)	C+, C−, P+, Pmid, P−, Q− (10–15)	S+ (16)

### 2.5 Judgement bias experiment

#### 2.5.1 Phases C and D (weeks 4 and 5; days 16–25)

The judgement bias testing phases followed the design used in Phase B with the addition of experimental probe trials on the Tuesday, Wednesday and Thursday of each week (Phase C testing days: 17–19; Phase D testing days: 22–24). An experimental probe trial was a trial on which the empty drinker was presented at one of the three intermediate locations. A daily testing session in Phase C comprised 16×30 s trials divided into two blocks. Block 1 comprised four reinforcement trials (2 sugar : 2 QHCl, pseudorandomized with two control trials (C+, C−) and three probe trials (P+, Pmid and P−). Block 2 comprised two control trials (C+, C−), pseudorandomized with three probe trials (P+, Pmid and P−), and two reinforcement trials (Q−, S+). Hamsters therefore had six experimental probe trials (two at each location: P+, Pmid and P−) in each daily session. Hamsters were removed as soon as they reached the empty drinker on all probe and control trials. Phase C comprised three testing days which generated six data points per probe location per hamster. On the Monday and Friday (days 16 and 20), hamsters received sessions as in Phase B. Phase D (week 5) was an exact repetition of Phase C, except that housing treatments were switched as explained below.

### 2.6 Housing treatments

At the start of the study hamsters were housed in their home cages provisioned with a thin layer of aspen chip substrate and lignocel bedding material (both from IPS, London, UK), a free-standing barred running wheel (diameter 15 cm) and two spirally wound cardboard tubes (20×10 cm). In the current study, emotion state was manipulated by adding or reducing enrichment. Choice of enrichment devices was informed by published work showing that their presence leads to changes in behaviour assumed to indicate preferences and improved welfare in hamsters [71–73]. Additional enrichment items comprised deeper aspen substrate and extra nesting material [71], two coloured transparent plastic huts (10×12 cm), a suspended hamster tent (15×12 cm), four hamster gnaw sticks and a wooden ledge (18×13 cm: all of which increase opportunity for natural and exploratory behaviour [72,73,77]). The metal barred wheel was replaced with a larger solid-floor plastic silent running ball-bearing wheel (16.5 cm, Silent Spinner Regular [83]).

Enrichment items that hamsters had not encountered previously (hamster gnaw sticks, tent, ledge and plastic running wheel) were added to all home cages on the Friday afternoon of Phase A (day 10 after training: figure 1). Animals were left to habituate to the novel enrichment devices over the weekend and throughout the next week (Phase B on the following Monday–Friday). Introducing novel enrichment items during training should reduce potentially confounding context-dependent learning effects on animals’ performance in the two treatments during later testing sessions [84]. After training on the Thursday of Phase B, the maximum complement of enrichment items was added to half of the cages (Treatment E: ‘enrichment added’) while the novel enrichment items were removed from the other half of the cages (Treatment R: ‘enrichment removed’). Discrimination training was then maintained on the Friday and the following Monday. This ensured all animals had undergone some form of ‘change’ in housing environment prior to the start of testing in Phase C). Testing in Phase C was conducted on three days (Tuesday–Thursday), which corresponded to the fifth, sixth and seventh days of having a full complement of enrichment devices added to the home cage for four sibling groups, and the fifth, sixth and seventh days following reduction in enrichment from the home cage for four sibling groups. Following the third day of testing in Phase C (Thursday), housing treatments were switched so that the hamsters receiving Treatment E now received Treatment R, and vice versa. Maintenance on the task following the procedure described for Phase C was conducted on the Friday and following Monday. Hamsters were then tested again in Phase D on the following Tuesday–Thursday.

### 2.7 Behavioural measures

Behaviour during experimental trials (probe and control trials on the Tuesday–Thursday in Phases C and D) was recorded to explore possible treatment-induced differences in arousal and stress-related activity. Arousal was assessed by calculating a ‘locomotion score’: the arena floor was marked out as a grid of 12 squares, and the number of squares the hamsters entered prior to reaching the drinker (or until the end of the 30 s trial on ‘no-go’ trials) was recorded. General stress-related activity was assessed by calculating an ‘anxiety score’: behaviours considered to indicate stress or anxiety in hamsters (autogrooming, attempting to climb arena walls to ‘escape’, freeze, stereotypy, rearing and sniffing [7,74,76,77]) were recorded using 1/0 scoring [85] (range 0–6) during each trial.

OF, LDE and neophobia tests were conducted during Phase C and D (figure 1). Each hamster took part in one 2 min trial on each of an OF and an LDE test on the Thursday afternoon after judgement bias testing in each phase. The OF apparatus measured 1 m^2^. Total number of squares entered and time spent in the middle square were recorded. In the LD emergence test, the hamster was placed in a wooden hut (15 cm^3^) and time taken to emerge into a well-lit exterior was recorded. Neophobia was tested on the Friday of Phase D. Each hamster took part in nine trials in the arena, during which the time to approach the sugar drinker when a novel object was next to the drinker (*n*=4 trials) or not present (*n*=5 trials) was recorded. A different novel object was used on each trial and objects varied in shape and colour and were unfamiliar to the hamsters.

### 2.8 Data analysis

Each hamster took part in a total of 60 experimental trials (30 in each housing treatment): 36 probe trials (12 at each of the three probe locations) and 24 control trials (12 at each of the C+ and C− locations). These were dispersed amongst a total of 36 reinforcement trials (Sugar and QHCl). A criterion of more than 50% correct responses (‘go’ to the C+ and ‘no-go’ to the C−) on control trials across testing sessions was set for each hamster to be included in the analyses. We used a generous criterion for inclusion in the analyses since we wanted to explore the potential influence of a range of factors on performance in the judgement bias task. We did not want to bias findings by selectively removing hamsters who were perceived to be poor performers if changes in performance reflected meaningful responses to the enrichment treatments. However, we wanted to remove hamsters who stopped responding on the task altogether.

#### 2.8.1 Model development

All recorded explanatory variables were considered for inclusion in the maximal model. These were: treatment (E, R), trial type (C+, P+, Pmid, P−, C−), testing block (blocks 1 and 2), year of testing (2011, 2013), day of testing (Tuesday, Wednesday or Thursday), order of testing (E first, R first), trial number (trial 1–16), experimenter ID, hamster ID (24 hamsters), sibling group (eight groups) and speed to learn the discrimination task (3–16 days). Preliminary assessment for colinearity between these variables was conducted in the statistical package R v. 3.0.1 [86]. Where two or more variables covaried, these were serially entered into a series of generalized linear models (GLMs) and the variable resulting in the GLM with the lowest AIC_c_ value was retained [80,81].

Preliminary analyses revealed that testing block correlated with trial number (*r*=0.63); block had the lowest AIC_c_ and was retained. Week of testing correlated strongly with day of testing (*r*>0.9); day was retained. Sibling group, year in which the data were collected, experimenter ID and speed to learn the discrimination task were correlated with each other (all *r*s>0.36); sibling group was retained. Order of testing was correlated with sibling group and hamster ID (as siblings were housed together and therefore underwent the same housing treatment together: *r*s>0.55). Because previous studies have found an effect of order on responses during judgement bias tests, we conducted a glm (order × treatment) to explore possible order effects. This revealed a significant order × treatment interaction (estimate=0.512, error=0.23, *z*=2.19, *p*=0.029). To control for this effect in the final model order of testing was retained with sibling group and male ID as nested random factors.

A maximal model was defined using the glmer package in R [86]. Go/no-go responses were the dependent binary variable. Treatment, trial type and block were the fixed factors; day of testing was included as a random factor and order of testing, sibling group and male ID were included as nested random factors. Treatment, trial type and block were also included as interaction terms:

$$\begin{array}{rl} & \text{glmer}(\text{NoGo}∼\text{Treatment}\times \text{Trial Type}\times \text{Block}+(1|\text{Day Test})+(1|\text{Order/Siblgrp/Male ID}),\\ & \text{family}=\text{binomial}). \end{array}$$

These explanatory variables were used to create a total of 15 candidate models all with a logit link function and a binomial error distribution (electronic supplementary material, table S1). Models were compared and selected using the AIC_c_, with the lowest AIC_c_ value indicating the best model fit. All models within 2 AIC_c_ of the best model were retained as the final subset of models [80,81].

To test for an effect of fixed factors featured in the best-fit model(s) at each of the probe and control locations separately, test models were created for each probe and control location (with random factors held constant), and with each fixed factor from the best model(s) included separately. Test models were then tested against the null model for each probe and control location using the anova function in R [86]. Criterion for significance was *p*<0.05.

An example of the null model used with data for trial location separately is as follows:

NoGo∼(1|Day Test)+(1|Order/Siblgrp/Male ID).

### 2.9 Behavioural measures

Behavioural data for hamsters while in the arena were analysed separately as they were collected on a subset of the experimental trials. For each hamster, the locomotion score was divided by the trial duration (latency to approach the drinker, or 30 s in the case of no-gos) to give a locomotion index for each trial. For behaviour in the arena, anxiety score was divided by the trial duration. Data were tested for normality and sqrt transformed where necessary. Locomotion and anxiety indices were then entered separately as dependent variables in the 15 candidate models and the best-fit models selected as described above.

LD emergence, OF and neophobia test data were also analysed separately as these data were collected at the end of each treatment week (Phases C and D—Phase D only for neophobia data). Owing to the small number of trials, these data were analysed using non-parametric Wilcoxon tests. Neophobia data were tested following a model selection procedure as outlined above using the lmer function in R [87]. Time to approach the drinker was the dependent variable (since hamsters approached the drinker on all novelty trials and most sugar trials), with fixed factors treatment (E or R) and trial type (sugar or sugar + novel) with nested random factors sibling group and hamster ID.

## 3. Results

### 3.1 Habituation

Animals defecated in the arena on the first day of familiarization, but not thereafter. Hamsters readily approached and drank from the drinker within each 5 min session.

### 3.2 Learning the discrimination task (days 1–16)

Performance data for hamsters during Phases A and B are given in the electronic supplementary material, table S2 and table S3. On average, hamsters reached criterion for learning the discrimination task in 8 days (79 trials; range 3–16 days. Eighteen hamsters reached criterion for learning the discrimination task before the end of Phase A (day 10). Five hamsters reached criterion before the end of Phase B (day 15). One hamster reached criterion on the first day in Phase C (day 16).

### 3.3 Performance on control trials in Phase B

Group-level accuracy for discrimination and control trials during phase B are given in the electronic supplementary material, table S3. Hamsters correctly approached the sugar location on 84% of trials and the sugar control (C+) on 71% of trials. They correctly did not approach the QHCl location on 64% of trials and the QHCl control (C−) on 69% of trials.

Maintenance of performance on the discrimination task during testing in Phases C and D: of the 30 hamsters that took part in testing, 24 hamsters (*n*=15 from 2011 and *n*=9 from 2013) from eight sibling groups performed to criterion on control trials during the judgement bias test in Phases C and D and were included in the final analysis. Performance data for these 24 are given in the electronic supplementary material, table S4. Of the six hamsters that were not included, one was removed from the study due to health reasons, and the remaining five failed to approach the C+ during the enrichment treatment (*n*=3) or during both treatments (*n*=2).

### 3.4 Model selection

The factors treatment, trial type and block of testing appeared in the best-fitting models, explaining 84% of the variance in the data (table 2).

**Table 2.** AIC_c_-ranked candidate model set showing the relative importance of fixed effects (treatment, trial type, block) and random effects (order of testing, sibling group, hamster ID and day) in explaining tendency to approach drinkers of known and uncertain reward and punishment value.

model fixed effects	random effects (/nested)	d.f.	log likelihood	AIC	delta	weight
treatment, trial × block	(order/sibling grp/ID), day	15	−772.932	1576.2	0.00	0.622
treatment, trial	(order/sibling grp/ID), day	11	−778.072	1578.3	2.11	0.216

### 3.5 Localizing significant effects

#### 3.5.1 Probe trials

**Figure 3.**
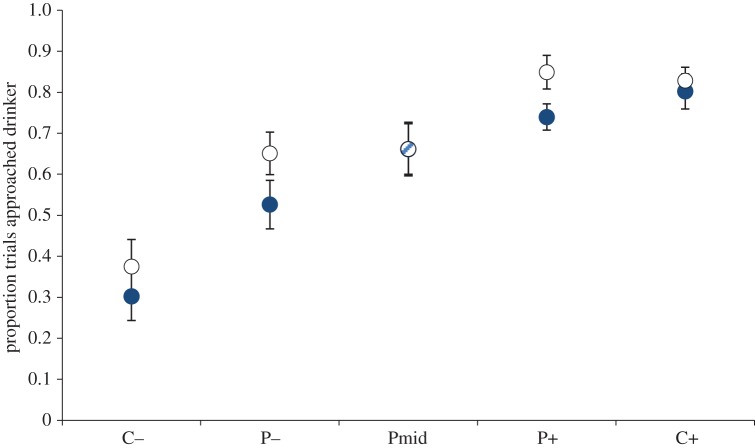
Mean proportion (± s.e.) of approaches to each of the probe and control locations when enrichment had been added (open circles) or reduced (closed circles). Hamsters approached the outer probes (P− and P+) significantly more often following addition of enrichment compared to when enrichment had been reduced. Hamsters approached the middle probe equally often in both treatments. CQHCl control; P− probe closest to the QHCl location; Pmid middle probe; P+ probe closest to the sugar location; C+ sugar control.

Anovas comparing the effect of treatment against the null model were conducted for each probe separately (table 3). These revealed a strong effect of treatment at the two outer probes, with no effect at the middle probe: hamsters approached the two outer probes more often in treatment E than R (figure 3). Comparison of the effect of block against the null model for each probe separately revealed a strong effect of block on responses to the Pmid and P+, but not the P−: hamsters made fewer approaches to the Pmid and P+ in the second compared to the first block.

**Table 3.** Comparison of test models (fixed factor of either treatment or block) with the null model for each of the three probes, two control locations and the reinforcing sugar or QHCl trials. Values for the null model are given in parentheses. Test models which were shown by ANOVA to deviate significantly from the null are indicated by asterisks.

trial	intercept	d.f.	log likelihood	AIC_c_	delta	weight
probe (+)						
treatment	−1.268	6	−143.38	299.1*	0.00	0.589
block	−2.743	6	−143.98	300.3*	1.19	0.325
null	(−0.879)	(5)	(−146.35)	(302.9)	(3.84)	(0.09)
probe (mid)						
treatment	−0.512	6	−166.48	345.3	6.37	0.036
block	−2.381	6	−163.30	338.9*	0.00	0.870
null	(−0.453)	(5)	(−166.58)	(343.4)	(4.46)	(0.094)
probe (−)						
treatment	−0.508	6	−173.17	358.7*	0.00	0.745
block	0.244	6	−175.46	363.3	4.59	0.075
null	(−0.202)	(5)	(−175.64)	(361.5)	(2.85)	(0.18)
control (+)						
treatment	−1.707	6	−129.28	270.9	0.25	0.40
block	−1.477	6	−130.20	272.7	2.10	0.16
null	(−1.484)	(5)	(−130.20)	(270.6)	(0.00)	(0.45)
control (−)						
treatment	0.571	6	−161.51	335.4	0.71	0.29
block	−0.130	6	161.49	335.3	0.66	0.30
null	(0.732)	(5)	(−162.20)	(334.6)	(0.00)	(0.41)
reinforcer (S+)						
treatment	−2.807	6	−138.62	289.4	9.32	0.00
block	−3.990	6	−133.96	280.1**	0.00	0.97
null	(−2.695)	(5)	(−138.84)	(287.8)	(7.70)	(0.02)
punisher (QHCl)						
treatment	0.509	6	−226.53	465.3	1.38	0.23
block	0.246	6	−226.25	464.7	0.82	0.31
null	(0.602)	(5)	(−226.88)	(463.9)	(0.00)	(0.46)

*p<0.05; **p<0.01.

#### 3.5.2 Control and reinforcement trials

There was no effect of treatment or block at either of the two control locations (or on the QHCl trials: table 3). There was a significant effect of block on approaches to the sugar drinker with hamsters approaching less often in the second block of testing compared to the first. Comparison of bottle weights revealed hamsters drank equivalent amounts of sugar water in the two treatments (*t*_15_=1.56,*p*=0.14).

### 3.6 Behavioural measures

Trial type explained more than 76% of the variance in locomotion in the arena (table 4). Hamsters locomoted more per second on sugar, C+ and P+ trials compared to the C− baseline (table 5). For anxiety index, no model explained the variance any better than the null model.

**Table 4.** Best-fit models for locomotion and anxiety indices for hamsters during test trials in the arena. All models within 2AIC_c_ of the best model are shown for each behavioural index.

model fixed effects	intercept	d.f.	log likelihood	AIC	delta	weight
locomotion index						
trial	0.347	12	−188.11	400.4**	0.00	0.762
null	(0.443)	(6)	(−255.45)	(523.0)	(122.51)	(0.000)
anxiety index						
block	0.2732	7	712.43	−1410.8	0.00	0.412
null	(0.3398)	(6)	(711.01)	(−1410.0)	(0.83)	(0.273)
block + treatment	0.2828	8	712.65	−1409.2	1.58	0.187

**p<0.01.

**Table 5.** The linear relationship between trial type and locomotion in the arena. Hamsters locomoted most per second on sugar trials and least on QHCl trials. Values calculated using C− used as reference.

locomotion	estimate	s.e.	*t*-value
intercept	0.35	0.09	3.91
sugar	0.22	0.02	8.17
C+	0.21	0.02	7.95
P+	0.18	0.02	6.74
Pmid	0.06	0.02	2.31
P−	0.02	0.02	0.67
QHCl	−0.01	0.02	−0.50

**Figure 4.**
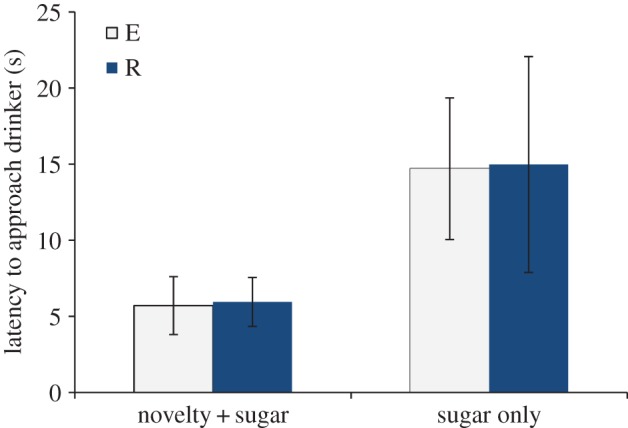
Mean latency (±s.e.) for hamsters to approach the sugar drinker with and without a novel object present. E, Enrichment added; R, Enrichment removed.

Non-parametric tests revealed no difference between treatments in performance in the OF (*n* squares entered: *W*=105, *p*=0.50; time in centre: *W*=129, *p*=0.95) or LDE (*W*=87, *p*=0.12). Model selection performed on data from neophobia tests revealed that trial type explained 70% of the variance in the data (all other models greater than 2 AIC_c_ points from best model). Hamsters were faster to approach the sugar drinker when there was a novel object situated next to it than when the sugar drinker alone was presented (figure 4). This was not affected by housing treatment.

## 4. Discussion

We developed a spatial judgement bias test for use with hamsters, using short-term changes in enrichment to validate the task. Firstly, hamsters learned to discriminate between drinkers at two locations: they were more likely to approach the positive location and less likely to approach the negative location. Therefore, the spatial judgement bias task is suitable for further development with hamsters. Secondly, hamsters were more likely to approach an empty drinker at ambiguous locations when enrichment had been added to their cage for the previous week, than when it had been removed. Hamsters approached more often as the probe neared the positive location. Importantly, there was no effect of treatment on approaches at the extreme cue locations. This difference in response to ambiguity is consistent with the judgement bias model of emotion–cognition interaction previously reported for a range of species [82]. This is the first study to report evidence for emotion-mediated judgement bias in a species of hamster.

Our unique pattern of findings has implications for continued refinement in the design of the judgement bias task by: (i) using a VRR during training to remove cueing effects of reinforcers; (ii) statistically controlling for potentially confounding effects by including additional factors (e.g. time, order of testing) as random effects in mixed models, and using behavioural measures to distinguish valenced judgements from arousal, risk-taking or perceptual factors; (iii) including a VRR during training, and control trials during testing to reduce rate of learning that probes were never rewarded; and (iv) using robust statistical approaches such as mixed effects models to control for potentially confounding factors in studies that often have small sample sizes. Our findings also have theoretical implications for: the value of cognitive measures of emotion over behavioural and experimental measures alone; the study of positive emotions; and further understanding the mechanisms underlying responses to ambiguous cues to reward and punishment.

### 4.1 Control trials and behavioural measures distinguish judgement bias from alternative explanations

Increased responding to two of the probes may be interpreted as valenced judgements of likelihood of reward or punishment because hamsters showed no difference between treatments in (i) proportion of approaches on control trials at the sugar drinker location and (ii) proportion of approaches on control trials at the QHCl location. If changes in responses were due to a generalized increase in arousal (or a shift in perception of the empty drinker, or risk-taking behaviour), then we would expect to see greater or fewer responses on the control trials as well as at the probes. Behavioural indices of arousal (locomotion and activity) collected during trials further support this interpretation. Hamsters showed no difference between treatments in rate of locomotion or anxiety-related behaviour during trials. Control trials and additional behavioural measures are therefore an effective way of controlling and accounting for possible arousal effects on responses.

### 4.2 Linear mixed effects models with random and nested factors provide robust analysis of data

The information-theoretic approach using generalized linear mixed models (GLMMs) provided a flexible and highly informative means of exploring our data. By testing the effect of fixed factors (here treatment and block) at each drinker location separately, we were able to tease apart the effects of different factors on responses at each probe. An advantage of the multi-model inference approach is that it provides one or more ‘best-fit’ models with information on the relative fit of these models to the spread of data. Using a hierarchical nested approach, we were able to control for a range of additional factors including individual differences in rate of learning, relatedness within sibling groups, order of testing, as well as for changes in performance over testing days. Using mixed effects models prevents problems of interpretation when there are temporal effects on performance (for example, in Scollo *et al.* [49] a shift in judgement bias was reported in pigs on the third day of testing, but no difference was found on previous days or when a mean across days was considered).

The results we report here meet the performance criteria suggested elsewhere for applying mixed models to judgement bias data [82], but we argue the model selection approach we present provides a more informative and intuitive analysis than that proposed by Gygax [82]. By testing our best-fit models against the null model at each probe location, we were able to identify the location of mood effects on responses to the probes. Probes are categorically different from the control locations (about which we have different predictions). The approach we advocate may be more useful for distinguishing the location of effects when identifying different emotion states, or processes involved in approach and avoidance (discussed below). Furthermore, a unified statistical approach to judgement bias studies would allow better comparison between studies and meta-analyses.

### 4.3 Learning and motivation may influence responses to ambiguous probes

In some previous studies, it has not been possible to rule out possible learning or motivational effects on responses to probes, especially if the effect of treatment is small [22,67,69], animals took part in a relatively large number of trials (more than 50 trials in some studies [26,44,62,65]) or where changes in responses at the learned locations were also detected (e.g. [7,30,32,39,41,43]). To reduce the likelihood, or speed, of learning that the probes were never rewarded, we used variable reinforcement during training. We then controlled for learning effects in the analyses by including day of testing as a random factor in our models. Block featured in the best model only in interaction with trial type. The main effect of block was found at the S+, P+ and Pmid (hamsters approached less often in the second of the two experimental blocks), but not at the other locations. There is therefore a possibility that learning may affect responses to ambiguous cues to reward, or that learning occurred across all locations but the lack of reduction in responses in the second block for either of the P−, C− or QHCl trials was concealed by a floor effect in the data. Alternatively, reduced responding to locations at the positive end may reflect reduced sugar motivation. In either case, any changes due to learning or motivation did not differ between treatments: hamsters drank equal amounts of sugar water during the two treatments, and there was no interaction of block with treatment. For hamsters, at least, it may therefore not be suitable to run more than one or two trials per probe location in a daily testing session. Including a measure of time (here, block and day) in analyses can provide a way of examining potential changes in responding over trials. This may be useful for refining protocols to optimize aspects of study design such as number and spacing of trials within and across days.

### 4.4 Not all probes are equal: the spatial judgement task may reveal qualitatively different emotion–cognition interactions

Our study presents a unique pattern of findings: a shift in bias at the two outer probes with no evidence of a bias at the middle probe. Changes in enrichment explained 59% and 76% of the variance in responses to the P+ and P−, respectively, yet treatment failed to explain variance at the middle probe. There are several possible reasons for the lack of bias at the middle probe: the middle probe block explained 87% of the variance which may suggest a greater influence of learning effects at this (perhaps more highly) ambiguous location; the middle probe, being truly ambiguous, may have induced a greater level of approach–avoidance conflict and anxiety which masked any other emotion effects arising from the housing treatments; or hamsters may process mildly ambiguous cues to punishment (P−) or reward (P+) differently from ambiguous cues with no partial negative or positive association.

Responses at the two outer probes have been proposed to distinguish ‘anxiety’ and ‘depression’ in animal models [9,39,41,62]. Humans suffering from anxiety have an increased expectation of negative events, while people suffering from depression have both an increased expectation of negative events and a reduced expectation of positive events [88]. In the animal judgement bias task, responses to the rewarded stimulus and adjacent probe are considered to tap into brain mechanisms associated with seeking, approaching and gaining reward; responses to the punished stimulus are considered to tap into brain mechanisms associated with avoidance of threat. Reinforcement sensitivity theory [89] identifies that emotional responses to stimuli arise from the interaction of three motivational systems sensitive to reward (dopaminergic and associated systems relating to positive emotions and approach), punishment (amygdala, anterior cingulate and serotonergic systems relating to negative emotions and avoidance) and reward–punishment conflict [90,91]. Different emotional and cognitive processes may underlie responses to the ambiguous probes, reflecting differences in degree of ambiguity, approach, avoidance and approach–avoidance anxiety. Studies involving genetic models [12,16,55] or pharmaceutical manipulation of brain systems [35,36,41,55,59,62] may elucidate the mechanisms driving responses on the judgement bias test. At the least, we suggest several probes should be included in the test when used with hamsters (cf. for example, single ambiguous probe used with rats [58,59,63] and pigs [48]), and each probe should be examined separately in analyses (cf. [36]): in the example we present here, this was done by testing against the null model at each probe location.

### 4.5 Optimistic judgement bias in hamsters?

Mapping shifts in judgement bias on to emotional ‘states’ [9] remains a challenge. Hamsters responded to all probes at least as often as expected by chance in both treatments; the most parsimonious explanation is therefore that the addition of extra cage enrichment leads to a more ‘optimistic’ judgement bias in the hamsters. Removal of the additional enrichment items (back to standard enrichment condition which hamsters were used to) resulted in a negative shift (possibly back to the standard baseline?). It is difficult to interpret animals as categorically ‘optimistic’ or ‘pessimistic’ as there is no reference baseline against which to assess this.

### 4.6 Judgement bias tests may be more sensitive to changes in emotion than traditional behavioural tests

Finally, while the judgement bias task revealed a change in responses to ambiguous cues to possible reward and possible punishment, we found no differences in responses between treatments on the additional standard behavioural tests. Hamsters performed similarly on the OF, LDE and neophobia tests in both housing treatments. In the latter test, hamsters were equally interested in investigating the novel objects in both treatments and were much faster on trials when a novel object was present than for sugar drinker only trials. There was therefore no difference in exploratory tendency or fear of new stimuli (e.g. a drinker spout appearing at a new location) between treatments. These findings lend weight to the argument that the judgement bias test may detect shifts in emotion state not discernable from, or apparently contra to, traditional behavioural measures alone ([7,13,27,29,34,51,70], cf. [24,40,49,66]); may detect shifts earlier (the housing treatments were one week in duration and therefore relatively short-term); or may detect these shifts at different points in the time course of emotional response (e.g. [32,51,58]).

This study adds to the rapidly expanding body of literature which supports the judgement bias task as a means of detecting affect-mediated changes in cognition in non-human animals. This literature shows that shifts in judgement bias may be detected following a range of affect manipulations in a range of species, now including hamsters; and that cognitive measures may detect shifts in emotion not apparent from behavioural observation or traditional behavioural tests of emotion alone. The unique pattern of results reported in this paper adds weight to suggestions that different underlying brain systems may contribute to differences in responses to ambiguous cues to reward or punishment that may have value in distinguishing between different, but similarly valenced, emotional states. We cannot say whether the hamsters in our study *felt* happy in their enriched housing, but the changes in cognitive processing of ambiguous cues certainly suggests enriched hamsters became more optimistic about the likelihood of future reward when faced with uncertain information. Judgement bias tasks present a unique and valuable approach to assessing emotion in laboratory rodents, including hamsters. Future development of these approaches (for example, incorporating automated systems, exploring long-term and developmental effects of captive animal husbandry) should lead to improved welfare assessment across species.

## Supplementary Material

ESM1 R code

## Supplementary Material

ESM2 Individual rates of learning the discrimination task

## Supplementary Material

ESM3 Performance on control trials and reinforced trials during Phase B with R code

## Supplementary Material

ESM4 Performance on control trials and reinforced trials during Phase C and D

## Supplementary Material

ESM5 Data

## Data accessibility

The datasets and R code supporting this article have been uploaded as part of the electronic supplementary material, tables S1–S5. R is a freely available statistical software package and is available to download at: http://www.r-project.org/.
