# Supplementary material for: Happy hamsters? Enrichment induces positive judgement bias for mildly (but not truly) ambiguous cues to reward and punishment in Mesocricetus auratus
Source: R Soc Open Sci. 2015 Jul 29;2(7):140399. doi: 10.1098/rsos.140399 (PMC4632568; doi:10.1098/rsos.140399)
Supplement: ESM1 R code [file rsos140399supp1.docx]

Supplementary material

15 candidate models

Null3<-glmer(NoGo ~ (1|DayTest) + (1|Order/Cage/MaleID), family = binomial, data = A24DATAHamster5xPC)

Null3

nM16<- glmer(NoGo ~ Treatment + (1|DayTest) + (1|Order/Cage/MaleID), family = binomial, data = A24DATAHamster5xPC)

nM16

nM13<- glmer(NoGo ~ TrialType + (1|DayTest) + (1|Order/Cage/MaleID), family = binomial, data = A24DATAHamster5xPC)

nM13

nM18<- glmer(NoGo ~ Block + (1|DayTest) + (1|Order/Cage/MaleID), family = binomial, data = A24DATAHamster5xPC)

nM18

nM8<- glmer(NoGo ~ Treatment + TrialType + (1|DayTest) + (1|Order/Cage/MaleID), family = binomial, data = A24DATAHamster5xPC)

nM8

nM15<- glmer(NoGo ~ Treatment + Block + (1|DayTest) + (1|Order/Cage/MaleID), family = binomial, data = A24DATAHamster5xPC)

nM15

MMax3<- glmer(NoGo ~ Treatment + TrialType + Block + (1|DayTest) +(1|Order/Cage/MaleID), family = binomial, data = A24DATAHamster5xPC)

MMax3

nM12<- glmer(NoGo ~ TrialType + Block + (1|DayTest) + (1|Order/Cage/MaleID), family = binomial, data = A24DATAHamster5xPC)

nM12

nM21<-glmer(NoGo ~ Treatment*TrialType + (1|DayTest) + (1|Order/Cage/MaleID), family = binomial, data = A24DATAHamster5xPC)

nM21

nM22<-glmer(NoGo ~ Treatment*Block + (1|DayTest) + (1|Order/Cage/MaleID), family = binomial, data = A24DATAHamster5xPC)

nM22

nM23<-glmer(NoGo ~ TrialType*Block + (1|DayTest) + (1|Order/Cage/MaleID), family = binomial, data = A24DATAHamster5xPC)

nM23

nM24<-glmer(NoGo ~ Treatment*TrialType*Block + (1|DayTest) + (1|Order/Cage/MaleID), family = binomial, data = A24DATAHamster5xPC)

nM24

nM25<- glmer(NoGo ~ Treatment*TrialType + Block + (1|DayTest) + (1|Order/Cage/MaleID), family = binomial, data = A24DATAHamster5xPC)

nM25

nM26<-glmer(NoGo ~ Treatment*Block + TrialType + (1|DayTest) + (1|Order/Cage/MaleID), family = binomial, data = A24DATAHamster5xPC)

nM26

nM27<-glmer(NoGo ~ TrialType*Block + Treatment + (1|DayTest) + (1|Order/Cage/MaleID), family = binomial, data = A24DATAHamster5xPC)

nM27
