# Supplementary material for: Happy hamsters? Enrichment induces positive judgement bias for mildly (but not truly) ambiguous cues to reward and punishment in Mesocricetus auratus
Source: R Soc Open Sci. 2015 Jul 29;2(7):140399. doi: 10.1098/rsos.140399 (PMC4632568; doi:10.1098/rsos.140399)
Supplement: ESM2 Individual rates of learning the discrimination task [file rsos140399supp2.docx]

Supplement 2. Discrimination training – Phases A and B

Table S1. Time to learn the discrimination task. Criterion for learning the discrimination task was average speed to approach the sugar location < average speed to approach the QHCl location on each of three consecutive days. Days were counted from the first discrimination trial in day 1 of Phase A (third day on which hamster reached criterion is shown in table). Twenty one hamsters reached criterion in Phase A (i.e. the three consecutive days occurred before day 10 of training). ^C^Five hamsters reached criterion in Phase B; ^D^ one hamster reached criterion on the Monday of Phase C (testing started on the Tuesday).

| Year | Cage | Hamster | N days to criterion | N trials to criterion |
| --- | --- | --- | --- | --- |
| 2011 | 1 | 1 | 9 | 96 |
| 2011 | 2 | 2 | 16 | 146^D^ |
| 2011 | 3 | 3 | 10 | 94 |
| 2011 | 3 | 4 | 6 | 58 |
| 2011 | 3 | 5 | 6 | 58 |
| 2011 | 4 | 6 | 14 | 126^C^ |
| 2011 | 4 | 7 | 7 | 68 |
| 2011 | 4 | 8 | 8 | 78 |
| 2011 | 4 | 9 | 15 | 136^C^ |
| 2013 | 5 | 10 | 3 | 28 |
| 2013 | 5 | 11 | 7 | 68 |
| 2013 | 5 | 12 | 7 | 68 |
| 2013 | 5 | 13 | 8 | 78 |
| 2013 | 6 | 14 | 4 | 38 |
| 2013 | 6 | 15 | 6 | 56 |
| 2013 | 6 | 16 | 4 | 38 |
| 2013 | 6 | 17 | 8 | 78 |
| 2013 | 7 | 18 | 11 | 102^C^ |
| 2013 | 7 | 19 | 15 | 138^C^ |
| 2013 | 7 | 20 | 4 | 38 |
| 2013 | 8 | 21 | 9 | 86 |
| 2013 | 8 | 22 | 11 | 102^C^ |
| 2013 | 8 | 23 | 6 | 56 |
| 2013 | 8 | 24 | 7 | 66 |
| Mean |  |  | **8.42** | **79.00** |
| SD |  |  | **3.78** | **32.96** |
