# Supplementary material for: Happy hamsters? Enrichment induces positive judgement bias for mildly (but not truly) ambiguous cues to reward and punishment in Mesocricetus auratus
Source: R Soc Open Sci. 2015 Jul 29;2(7):140399. doi: 10.1098/rsos.140399 (PMC4632568; doi:10.1098/rsos.140399)

Supplement 3. Discrimination performance on control trials during Phase B

Table S2.1. Performance on the discrimination task for control trials was assessed by looking at difference in speed to approach the empty drinker at the sugar and QHCl locations. Hamsters were significantly faster to approach the sugar control (C+: mean=14.76 sec +- 5.61) than they were to approach the QHCl control (C-: mean=24.93sec +- 3.41; Wilcoxon matched-pairs test: W =41, P<< 0.001). Hamsters were significantly faster to approach the sugar drinker (mean=12.41sec +- 5.20) than they were to approach the QHCl drinker (mean=24.17sec +- 2.33; Wilcoxon matched-pairs test: W = 6, P<< 0.001).

| Year | Cage | ID | QHCL | C- | C+ | Sugar |  | Diff  QHCl - Sugar | Diff  C- - C+ |
| --- | --- | --- | --- | --- | --- | --- | --- | --- | --- |
| 11 | 1 | 1 | 25.11 | 26.23 | 12.20 | 13.83 |  | 11.28 | 14.03 |
| 11 | 2 | 2 | 25.22 | 27.97 | 26.87 | 20.78 |  | 4.44 | 1.10 |
| 11 | 3 | 3 | 19.20 | 12.35 | 7.91 | 7.66 |  | 11.55 | 4.44 |
| 11 | 3 | 4 | 22.57 | 24.13 | 17.27 | 15.45 |  | 7.12 | 6.86 |
| 11 | 3 | 5 | 25.08 | 24.54 | 6.23 | 5.74 |  | 19.34 | 18.32 |
| 11 | 4 | 6 | 24.60 | 26.38 | 12.21 | 8.56 |  | 16.04 | 14.17 |
| 11 | 4 | 7 | 24.35 | 22.64 | 6.77 | 6.09 |  | 18.26 | 15.87 |
| 11 | 4 | 8 | 24.93 | 25.11 | 12.92 | 10.20 |  | 14.74 | 12.19 |
| 11 | 4 | 9 | 24.21 | 26.04 | 9.93 | 10.35 |  | 13.86 | 16.11 |
| 13 | 5 | 10 | 16.48 | 20.08 | 6.44 | 4.47 |  | 12.00 | 13.64 |
| 13 | 5 | 11 | 24.75 | 25.39 | 16.46 | 15.30 |  | 9.44 | 8.93 |
| 13 | 5 | 12 | 24.30 | 22.49 | 20.62 | 19.85 |  | 4.45 | 1.87 |
| 13 | 5 | 13 | 26.24 | 24.96 | 14.57 | 15.92 |  | 10.31 | 10.39 |
| 13 | 6 | 14 | 23.94 | 25.54 | 7.59 | 7.85 |  | 16.09 | 17.95 |
| 13 | 6 | 15 | 24.34 | 24.59 | 15.01 | 14.16 |  | 10.18 | 9.58 |
| 13 | 6 | 16 | 26.53 | 26.80 | 14.71 | 13.55 |  | 12.98 | 12.09 |
| 13 | 6 | 17 | 26.61 | 27.03 | 10.05 | 13.52 |  | 13.09 | 16.98 |
| 13 | 7 | 18 | 23.61 | 29.27 | 19.97 | 13.00 |  | 10.61 | 9.30 |
| 13 | 7 | 19 | 25.50 | 28.17 | 17.97 | 9.78 |  | 15.72 | 10.20 |
| 13 | 7 | 20 | 23.61 | 25.80 | 16.17 | 8.67 |  | 14.94 | 9.63 |
| 13 | 8 | 21 | 21.22 | 25.00 | 16.50 | 11.72 |  | 9.50 | 8.50 |
| 13 | 8 | 22 | 26.89 | 26.53 | 21.80 | 18.61 |  | 8.28 | 4.73 |
| 13 | 8 | 23 | 26.61 | 29.43 | 21.43 | 14.39 |  | 12.22 | 8.00 |
| 13 | 8 | 24 | 24.17 | 21.77 | 22.70 | 18.33 |  | 5.83 | -0.93 |
| Mean |  |  | **24.17** | **24.93** | **14.76** | **12.41** |  | **11.76** | **13.58** |
| SD |  |  | 2.33 | 3.41 | 5.61 | 4.46 |  | 3.94 | 5.20 |

Table S2.2. Relative performance on discrimination trials for the sugar and QHCl, and their corresponding controls (C+ and C- respectively). Difference scores for proportion of non-approaches to the QHCl location compared to the sugar location during the last week of discrimination training (Phase C) are shown. Significantly more No-Gos were recorded on QHCl trials (mean=0.64 +- 0.12) than sugar trials (mean=0.16 +- 0.11; Wilcoxon matched-pairs test: W = 41, P<< 0.001). A similar pattern was shown for control trials (QHCl control mean=0.69 +- 0.12; Sugar control C+ mean: 0.29 +- 0.19); Wilcoxon matched-pairs test: W =41, P<< 0.001).

| Year | Cage | ID | QHCL | C- | C+ | Sugar |  | Diff  QHCl - Sugar | Diff  C- - C+ |
| --- | --- | --- | --- | --- | --- | --- | --- | --- | --- |
| 11 | 1 | 1 | 0.50 | 0.67 | 0.17 | 0.22 |  | 0.28 | 0.50 |
| 11 | 2 | 2 | 0.61 | 0.83 | 0.80 | 0.33 |  | 0.28 | 0.03 |
| 11 | 3 | 3 | 0.56 | 0.93 | 0.43 | 0.11 |  | 0.44 | 0.50 |
| 11 | 3 | 4 | 0.78 | 0.87 | 0.43 | 0.11 |  | 0.67 | 0.43 |
| 11 | 3 | 5 | 0.67 | 0.70 | 0.30 | 0.06 |  | 0.61 | 0.40 |
| 11 | 4 | 6 | 0.61 | 0.57 | 0.43 | 0.11 |  | 0.50 | 0.13 |
| 11 | 4 | 7 | 0.78 | 0.83 | 0.43 | 0.33 |  | 0.44 | 0.40 |
| 11 | 4 | 8 | 0.61 | 0.97 | 0.53 | 0.11 |  | 0.50 | 0.43 |
| 11 | 4 | 9 | 0.72 | 0.53 | 0.57 | 0.33 |  | 0.39 | -0.03 |
| 13 | 1 | 10 | 0.50 | 0.18 | 0.03 | 0.03 |  | 0.47 | 0.15 |
| 13 | 1 | 11 | 0.53 | 0.74 | 0.41 | 0.17 |  | 0.37 | 0.32 |
| 13 | 1 | 12 | 0.77 | 0.71 | 0.03 | 0.07 |  | 0.70 | 0.68 |
| 13 | 1 | 13 | 0.63 | 0.76 | 0.18 | 0.07 |  | 0.57 | 0.59 |
| 13 | 2 | 14 | 0.60 | 0.56 | 0.06 | 0.03 |  | 0.57 | 0.50 |
| 13 | 2 | 15 | 0.73 | 0.68 | 0.15 | 0.07 |  | 0.67 | 0.53 |
| 13 | 2 | 16 | 0.70 | 0.74 | 0.12 | 0.13 |  | 0.57 | 0.62 |
| 13 | 2 | 17 | 0.27 | 0.44 | 0.09 | 0.03 |  | 0.23 | 0.35 |
| 13 | 3 | 18 | 0.63 | 0.59 | 0.29 | 0.27 |  | 0.37 | 0.29 |
| 13 | 3 | 19 | 0.63 | 0.53 | 0.47 | 0.40 |  | 0.23 | 0.06 |
| 13 | 3 | 20 | 0.67 | 0.76 | 0.24 | 0.20 |  | 0.47 | 0.53 |
| 13 | 4 | 21 | 0.67 | 0.76 | 0.06 | 0.03 |  | 0.63 | 0.71 |
| 13 | 4 | 22 | 0.73 | 0.68 | 0.24 | 0.20 |  | 0.53 | 0.44 |
| 13 | 4 | 23 | 0.80 | 0.74 | 0.29 | 0.13 |  | 0.67 | 0.44 |
| 13 | 4 | 24 | 0.77 | 0.82 | 0.21 | 0.20 |  | 0.57 | 0.62 |
| Mean |  |  | **0.64** | **0.69** | **0.29** | **0.16** |  | **0.49** | **0.40** |
| SD |  |  | 0.12 | 0.17 | 0.19 | 0.11 |  | 0.14 | 0.20 |

R code for Wilcoxon test to compare time to approach sugar and QHCl locations (including controls), to accompany Table S1.1:

> wilcox.test(datT$Sugar, datT$QHCL, paired=T,conf.int=T)

Wilcoxon rank sum test with continuity correction

data: datT$Sugar and datT$QHCL

W = 6, p-value = 6.452e-09

alternative hypothesis: true location shift is not equal to 0

95 percent confidence interval:

-13.50000 -10.08333

sample estimates:

(pseudo)median

-11.78694

> wilcox.test(datT$SC, datT$QC, paired=T,conf.int=T )

Wilcoxon rank sum test

data: datT$SC and datT$QC

W = 41, p-value = 1.57e-08

alternative hypothesis: true location shift is not equal to 0

95 percent confidence interval:

-12.737794 -8.021127

sample estimates:

(pseudo)median

-10.28277

R code for wilcoxon test to compare proportion of responses on Sugar v QHCl trials, to accompany Table S1.2:

> wilcox.test(dat$Sugar, dat$QHCl, paired=T,conf.int=T)

Wilcoxon signed rank test with continuity correction

data: dat$QHCL and dat$Sugar

V = 300, p-value = 1.935e-05

alternative hypothesis: true location shift is not equal to 0

95 percent confidence interval:

0.4250200 0.5600742

sample estimates:

(pseudo)median

0.490025

> wilcox.test(dat$C., dat$C..1, paired=T,conf.int=T)

Wilcoxon signed rank test with continuity correction

data: dat$C. and dat$C..1

V = 298, p-value = 2.494e-05

alternative hypothesis: true location shift is not equal to 0

95 percent confidence interval:

0.3150481 0.5049697

sample estimates:

(pseudo)median

0.423419

Figure S1.1 Histograms showing the frequency of NoGo responses (y axis) during the final phase of discrimination training (Phase B) for the 24 hamsters which took part in testing during Phase C and D. On the x axis 0.0 signifies hamsters approached on all trials. 1.0 signifies hamsters did not approach on all trials. a) sugar trials; b) QHCl trials; c) control trials at sugar location; d) control tirlas at QHCl location.

1. b)


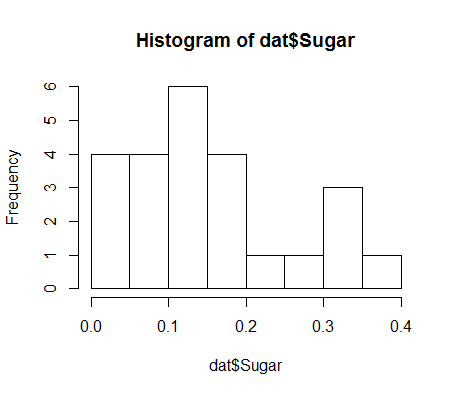

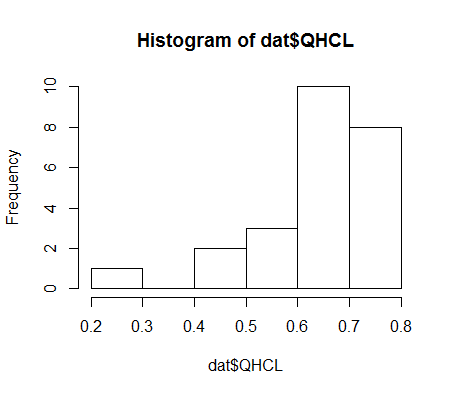


1. d)


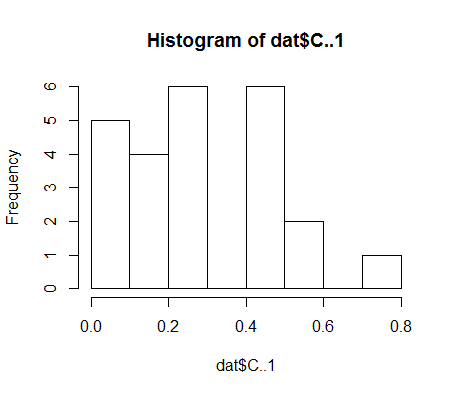

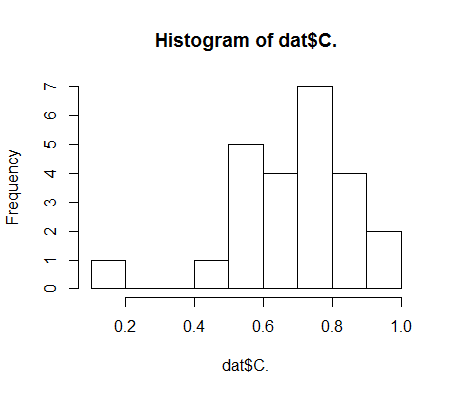

Supplement: ESM3 Performance on control trials and reinforced trials during Phase B with R code [file rsos140399supp3.docx]
