# Supplementary material for: Happy hamsters? Enrichment induces positive judgement bias for mildly (but not truly) ambiguous cues to reward and punishment in Mesocricetus auratus
Source: R Soc Open Sci. 2015 Jul 29;2(7):140399. doi: 10.1098/rsos.140399 (PMC4632568; doi:10.1098/rsos.140399)
Supplement: ESM4 Performance on control trials and reinforced trials during Phase C and D [file rsos140399supp4.docx]

Supplement 3. Performance on discrimination task during judgement bias testing (Phase C & D)

Table S3.1. Performance (Time in seconds to approach each drinker) on the discrimination task when hamsters (n=24) had extra enrichment in the home cages. Positive difference scores indicate hamster ran more slowly

| Year | Order | Cage | ID | QHCl | C- | C+ | Sugar |  | Diff  QHCl - Sugar | Diff  C- - C+ |
| --- | --- | --- | --- | --- | --- | --- | --- | --- | --- | --- |
| 11 | 1 | 1 | 1 | 15.75 | 20.75 | 8.00 | 6.25 |  | 9.50 | 12.75 |
| 11 | 1 | 2 | 2 | 22.50 | 23.50 | 15.25 | 9.50 |  | 13.00 | 8.25 |
| 11 | 2 | 3 | 3 | 21.40 | 22.20 | 10.80 | 17.40 |  | 4.00 | 11.40 |
| 11 | 2 | 3 | 4 | 20.40 | 26.60 | 15.20 | 6.20 |  | 14.20 | 11.40 |
| 11 | 2 | 3 | 5 | 26.20 | 25.00 | 9.20 | 14.40 |  | 11.80 | 15.80 |
| 11 | 2 | 4 | 6 | 26.00 | 30.00 | 16.00 | 8.30 |  | 17.70 | 14.00 |
| 11 | 2 | 4 | 7 | 27.80 | 28.00 | 14.20 | 19.50 |  | 8.30 | 13.80 |
| 11 | 2 | 4 | 8 | 30.00 | 30.00 | 15.60 | 5.10 |  | 24.90 | 14.40 |
| 11 | 2 | 4 | 9 | 15.80 | 25.00 | 9.80 | 9.50 |  | 6.30 | 15.20 |
| 13 | 1 | 5 | 10 | 18.61 | 14.42 | 4.17 | 6.20 |  | 12.40 | 10.25 |
| 13 | 1 | 5 | 11 | 17.21 | 18.87 | 16.55 | 11.33 |  | 5.88 | 2.32 |
| 13 | 1 | 5 | 12 | 22.70 | 26.01 | 9.03 | 5.61 |  | 17.09 | 16.98 |
| 13 | 1 | 5 | 13 | 26.91 | 26.65 | 9.04 | 9.22 |  | 17.69 | 17.61 |
| 13 | 1 | 6 | 14 | 25.07 | 19.40 | 7.47 | 2.66 |  | 22.41 | 11.93 |
| 13 | 1 | 6 | 15 | 25.60 | 27.85 | 8.10 | 6.07 |  | 19.53 | 19.75 |
| 13 | 1 | 6 | 16 | 22.32 | 17.35 | 11.76 | 14.00 |  | 8.32 | 5.59 |
| 13 | 1 | 6 | 17 | 19.63 | 20.21 | 8.23 | 6.22 |  | 13.41 | 11.97 |
| 13 | 2 | 7 | 18 | 28.19 | 23.39 | 13.25 | 8.92 |  | 19.26 | 10.14 |
| 13 | 2 | 7 | 19 | 30.00 | 22.88 | 19.78 | 11.71 |  | 18.29 | 3.11 |
| 13 | 2 | 7 | 20 | 21.38 | 30.00 | 10.09 | 10.12 |  | 11.26 | 19.91 |
| 13 | 2 | 8 | 21 | 29.27 | 28.59 | 12.47 | 8.83 |  | 20.45 | 16.12 |
| 13 | 2 | 8 | 22 | 24.82 | 30.00 | 19.43 | 10.19 |  | 14.63 | 10.57 |
| 13 | 2 | 8 | 23 | 25.80 | 29.54 | 9.55 | 6.56 |  | 19.24 | 19.99 |
| 13 | 2 | 8 | 24 | 29.45 | 22.40 | 18.39 | 15.69 |  | 13.77 | 4.01 |
| Mean |  |  |  | 23.87 | 24.53 | 12.14 | 9.56 |  | 14.30 | 12.39 |
| SD |  |  |  | 4.32 | 4.36 | 4.09 | 4.09 |  | 5.39 | 4.98 |

Table S3.2. Performance (Time in seconds to approach each drinker) on the discrimination task when hamsters (n=24) had received a reduction in the enrichment in the home cages.

| Year | Order | Cage | ID | QHCl | C- | C+ | Sugar |  | Diff  QHCl - Sugar | Diff  C- - C+ |
| --- | --- | --- | --- | --- | --- | --- | --- | --- | --- | --- |
| 11 | 1 | 1 | 1 | 22.60 | 29.80 | 14.00 | 6.10 |  | 16.50 | 15.80 |
| 11 | 1 | 2 | 2 | 21.00 | 26.40 | 10.40 | 8.10 |  | 12.90 | 16.00 |
| 11 | 2 | 3 | 3 | 27.25 | 27.25 | 22.75 | 5.88 |  | 21.38 | 4.50 |
| 11 | 2 | 3 | 4 | 19.00 | 28.75 | 7.50 | 5.75 |  | 13.25 | 21.25 |
| 11 | 2 | 3 | 5 | 24.75 | 24.75 | 15.25 | 8.13 |  | 16.63 | 9.50 |
| 11 | 2 | 4 | 6 | 29.75 | 15.50 | 11.00 | 11.50 |  | 18.25 | 4.50 |
| 11 | 2 | 4 | 7 | 30.00 | 30.00 | 18.00 | 20.00 |  | 10.00 | 12.00 |
| 11 | 2 | 4 | 8 | 20.50 | 20.25 | 10.00 | 8.38 |  | 12.13 | 10.25 |
| 11 | 2 | 4 | 9 | 25.75 | 25.75 | 25.25 | 19.13 |  | 6.63 | 0.50 |
| 13 | 1 | 5 | 10 | 14.41 | 11.73 | 11.56 | 5.63 |  | 8.78 | 0.17 |
| 13 | 1 | 5 | 11 | 26.72 | 28.81 | 9.55 | 10.32 |  | 16.40 | 19.26 |
| 13 | 1 | 5 | 12 | 22.94 | 30.00 | 4.59 | 3.70 |  | 19.24 | 25.41 |
| 13 | 1 | 5 | 13 | 25.61 | 30.00 | 8.72 | 9.40 |  | 16.22 | 21.28 |
| 13 | 1 | 6 | 14 | 28.41 | 28.87 | 2.95 | 2.83 |  | 25.58 | 25.92 |
| 13 | 1 | 6 | 15 | 26.78 | 20.61 | 5.51 | 6.78 |  | 20.00 | 15.10 |
| 13 | 1 | 6 | 16 | 28.67 | 30.00 | 7.28 | 8.17 |  | 20.50 | 22.72 |
| 13 | 1 | 6 | 17 | 26.40 | 26.14 | 10.87 | 5.57 |  | 20.82 | 15.27 |
| 13 | 2 | 7 | 18 | 25.60 | 23.03 | 15.84 | 14.58 |  | 11.01 | 7.19 |
| 13 | 2 | 7 | 19 | 27.55 | 26.38 | 19.63 | 22.90 |  | 4.64 | 6.76 |
| 13 | 2 | 7 | 20 | 25.11 | 23.48 | 25.15 | 17.59 |  | 7.52 | -1.67 |
| 13 | 2 | 8 | 21 | 23.00 | 27.46 | 14.59 | 4.79 |  | 18.21 | 12.87 |
| 13 | 2 | 8 | 22 | 25.63 | 21.89 | 13.79 | 11.69 |  | 13.93 | 8.10 |
| 13 | 2 | 8 | 23 | 16.54 | 26.19 | 9.60 | 7.13 |  | 9.41 | 16.59 |
| 13 | 2 | 8 | 24 | 25.14 | 22.31 | 17.82 | 13.00 |  | 12.14 | 4.49 |
| Mean |  |  |  | 24.55 | 25.22 | 12.98 | 9.88 |  | 14.67 | 12.24 |
| SD |  |  |  | 3.87 | 4.63 | 5.99 | 5.33 |  | 5.23 | 7.87 |
